# Sub-fecundity and associated factors among mothers with natural planned conception attending antenatal care service in Arba Minch Health Facilities

**DOI:** 10.1371/journal.pone.0241995

**Published:** 2020-11-05

**Authors:** Jira Wakoya Feyisa, Sultan Hussen Hebo, Firdawek Getahun Negash, Negussie Boti Sidamo, Kabtamu Tolosie Gergiso, Mulugeta Shegaze Shimbre, Bitew Mekonnen Chekol

**Affiliations:** 1 Department of Public Health, Faculty of Medical and Health Sciences, Mettu University, Mettu, Ethiopia; 2 School of Public Health, College of Medicine & Health Sciences, Arba Minch University, Arba Minch, Ethiopia; 3 Department of Statistics, College of Natural Sciences, Arba Minch University, Arba Minch, Ethiopia; 4 School of Nursing, College of Medicine & Health Sciences, Arba Minch University, Arba Minch, Ethiopia; University of Mississippi Medical Center, UNITED STATES

## Abstract

**Background:**

Fecundity is a physiological ability to have children. The inability to get the desired child which was commonly caused by the prolonged time to conceive due to unwanted non-conception period increased from time to time. As a result, many couples are developing psychological, social, and economic problems and unstable life. However, information on fecundity status is limited in Ethiopia context. Therefore, this study aimed to assess the proportion of sub-fecundity and associated factors in Ethiopia context.

**Methods:**

A health institution based cross-sectional study was conducted in Arba Minch health facilities from March 25 to April 25, 2020. By using a systematic sampling method, 539 mothers were selected for the study. Structured questionnaire was used for data collection. A binary logistic regression model was used to identify factors associated with the sub-fecundity. Variables with p-value <0.25 in the bi-variable logistic regression analysis were interred and checked for association in a multivariable logistic regression model. The level of statistical significance was declared at p-value <0.05.

**Result:**

The proportion of sub-fecundity was 17.8% with 95%CI (14.8%-21.3%). Mothers’ age ≥ 30 (AOR = 2.54, 95%CI; 1.18–5.48), partners’ age ≥ 35 (AOR = 2.20, 95%CI; 1.01–4.75), coffee consumption of ≥ 4 cups/day (AOR = 2.93, 95%CI; 1.14–7.53), menses irregularity (AOR = 3.79 95%CI; 2.01–7.14) and coital frequency of 1day/week (AOR = 3.65, 95%CI; 1.47–9.05) were significantly associated with the sub-fecundity.

**Conclusion:**

This study found that a substantial proportion of mothers were sub-fecund. Factors that contributed to the sub-fecundity were pre-pregnancy; mothers’ age, partners’ age, coffee drinking of ≥ 4 cups/day, coital frequency of 1day/week, and menses irregularity. Thus, efforts to prevent sub-fecundity should focus on awareness creation as to plan to conceive at early age, reducing coffee consumption, increasing days of coital frequency per week, and investigating and treating mothers with irregular menses.

## Introduction

In demography, the concept of fecundity is considered as a physiological ability to have children during the reproductive period while fertility is the actual delivery of a live birth. Fecundity and fertility are sometimes used interchangeably, because fecundity plays a role in fertility but they are referred to as different concepts [1, 2]. Fecundability is a probability to conceive per menstruation cycles that is reflected (measured) by waiting time to pregnancy (WTTP) which indicates the capacity to reproduce [2, 3]. Waiting time to pregnancy is the number of months or years the reproductive age women wait for conception; the number of waiting for cycles to conceive with unprotected sexual intercourse in the fertile phase of the menstrual cycles [4, 5].

The waiting time to conceive varies among reproductive-age women and reflects the variation of fecundity [6]. Waiting time of becoming pregnant with a natural plan to conceive may take months/years [7, 8]. An increased WTTP reflects decreased fecundity [9]. International Committee for Monitoring Assisted Reproductive Technology (ICMART) and the World Health Organization (WHO) describe failure to achieve pregnancy after exposed to unprotected sexual intercourse as a reproductive health problem when the time to conceive is greater than 12 months of WTTP [10]. Furthermore, epidemiological studies classify the fecundity status by using the waiting time to pregnancy as fecundity (within 12 cycles/months) and sub-fecundity (after 12 months of waiting time to conceive) [8, 11, 12].

Worldwide, the inability to get the desired child in 2009–2015 was ranging from 60 to 80 million [13, 14]. Demographic and reproductive health survey of 190 surveillance nations indicated that an inability to get the desired child which was commonly caused by the prolonged time to conceive due to unwanted non-conception period increased from 42 million in 1990 to 48.5 million in 2010 [15]. Studies in European countries also indicated that waiting time to pregnancy greater than 12 months ranges from 10.4% - 17.58% [16, 17]. The 2017 low to middle-income countries demographic and health survey report showed that sub-fecundity was 31.1% [18]. The 2016 South African report of waiting time to conceive more than 12 months was 22% [3]. In 2018, a study finding from Addis Ababa Ethiopia showed that sub-fecundity was 18.3% [8].

Sub-fecundity is the frequent health problem to partners living together [19]. Failed to achieve pregnancy after waiting for months/years create a severe issues among couples. As a result, couples exposed to sexually transmitted infections (STIs), divorce or separation, helplessness, psychological distress, social stigmatization, anxiety and economical problems [20]. Furthermore, after a prolonged time to conceive, some women may become pregnant that leads to an adverse birth outcome [21].

Evidence showed that different factors are related to sub-fecundity. Socio-demographic factors such as the age of couples [22, 23], abortion, the regularity of menstrual cycle and the use of contraceptive methods are identified as factors related to sub-fecundity [24, 25]. Recreational substances like; smoking cigarettes alcohol, chewing khat, and coffee drinking are also identified as factors related to sub-fecundity [8, 26–28].

Though identification of sub-fecundity and associated factors is very crutial for prevention, treatment and counseling purposes of reproductive age women with sub-fecundity in any setting [29], there is a limitation of the information in Ethiopia and other sub-Saharan African countries as far as our searching engine. Evidence based information on sub-fecundity has a siginificant contribution for anticipatory guidance for health care providers to improve understanding of preventable and unpreventable factors of sub-fecundity; guidance on actions that individuals and couples could take to address preventable factors of sub-fecundity. Furthermore, the information have an implication to shorten the waiting for time to pregnancy and reduce the cost of treatment for many cohabiting couples.

Therefore, this study was aimed to investigate sub-fecundity by using WTTP through identifying the proportion of sub-fecundity and associated factors among mothers with natural planned conception who are attending antenatal care (ANC) service in Arba Minch health facilities (AMHF).

## Materials and methods

### Study design and setting

A health institution based cross-sectional study was conducted in Arba Minch health facilities which are located in Arba Minch town, Gamo zone of Ethiopia. The town is 500 km away from Addis Ababa, the capital city of Ethiopia and 275 km from Hawassa, the capital city of SNNP region. The town has one government hospital, three health centers and 36 private clinics. The private clinics and Woze health center did not give ANC service during the study period. Other than this, Arba Minch health facilities are giving services for a total of 2,007,143 populations in a catchment area. The AMHFs are also providing maternal health care like ANC service for a total of 1640 mothers per month.

### Population

All pregnant mothers with natural planned conception who were attending antenatal care service at AMHFs were source population where as pregnant mothers with natural planned conception who were attending ANC service at AMHFs during the study period were study population for this study.

All pregnant mothers with a natural planned conception were included in this study. However, pregnant mothers with a diagnostic history of STIs and cervical cancer were excluded from this study since the mothers with such problems are commonly losing their physiological ability to conceive.

### Sample size determination

The sample size for this study was determined based on the objective of the study. For the first objective we used single population proportion formula. The following assumptions were considered: P(proportion of waiting time to pregnancy >12 months) = 18.3% which is taken from the study conducted in Addis Ababa [8].

$$n=\frac{{\left(Z(1-\frac{\alpha }{2})\right)}^{2}\times p\times (1-p)}{d^{2}}=\frac{{(1.96}^{2})\times (0.183\times 0.817)}{0.04^{2}}=359.$$

Where: n = required sample size; d = margin of error which is 4%; P = proportion of time to pregnancy >12 months = 0.183; Zα_/2_ = critical value for 95% confidence level which equals to 1.96. Thus, by considering 10% non-response rate the final sample size was 395 mothers.

The sample size for the second objective was determine by using two population proportion formula using Epi-Info-7.2 Stat Calc software. The following assumptions were considered: 95% confidence interval for a two-sided test; 80% power with a minimum detectable alternative of ± 5%; ratio of unexposed to exposed of 1:1; the proportion of pregnant mothers with irregular menses during pre-pregnancy (exposed group) = 73% and the proportion of pregnant mothers with regular menses during pre-pregnancy (non-exposed group) = 27%. Accordingly, the calculated sample size was 490 mothers. After consideration of 10% non-response rate the sample size become 539 pregnant mothers. Thus, the sample size calculated from second objective was larger than the sample size for the first objective. Therfore, the final sample size for this study was 539 pregnant mothers.

### Sampling technique

A systematic sampling technique was used to recruit the predetermined sample size (539). Before the selection of the study participants, proportion to size allocations of the sample size for the number of pregnant mothers in each health facilities was done. Finally, the required number (n = 539) of target women were selected using systematic sampling technique by using interval (*k*^th^ = 2) calculated from the monthly flow of pregnant mothers divided for the number of allocated sample to the health facilities which was 2 (every other) mothers with natural planned conception.

### Definitions and measurement

**Waiting time to pregnancy** was calculated from the starting time of unprotected sexual practice to the last menstrual period and subtracting months of sexual abstinence due to illness, movement of one partner from each other for work, or other causes. Waiting time to pregnancy was checked based on the question that asked the number of months or years that took a woman to become pregnant. The question was “How many months were you having sexual intercourse without doing anything to avoid pregnancy?” In this study, waiting time to pregnancy was used to identify fecundity status of mothers with natural planned conception. Waiting time to pregnancy was classified as dichotomies, sub-fecundity (greater than 12 months of waiting time to pregnancy) and fecundity (waiting time to pregnancy for 12 and below months) [10, 30].

**Fecundity status** is a physiological (biological) ability to reproduce which is the period between menarche and menopause in women [1].

#### Fertility

It refers to the actual production of live birth, output, or production of reproduction rather than the ability to have children. Demographers defined fertility as the reference of live birth, not stillbirth or abortion [1, 3].

#### Natural conception

Pregnancy without Assisted Reproductive Technology (ART), a biological/physiological processes.

#### Planned pregnancy

Indicate that if the mother intended to become pregnant, stopped contraception, her partner agrees and seek to become pregnant within preferred time with the schedule of couples to get conception before pregnancy [31].

### Data collection tool and procedure

A structured questionnaire for data collection was developed based on peer-reviewed literature [3, 8, 25]. After careful and detail reviews of available information, the questions for this study were extracted by the investigators. Then, each question was assessed carefully by the experts in the field of sexual and reproductive health. The final version of the questionnaire was prepared after pretesting on similar population attending the ANC service at neighboring public health facility. Before data collection pregnant mothers with unprotected sexual intercourse before the current pregnancy, free from any contraceptive method, intended to become pregnant as well as agreed with a partner according to schedule before conception for pregnancy were differentiated whether the pregnancy was planned or not planned [31]. Then data were collected by eight BSc. holder females midwives after the ANC service was given for the mother using the pre-tested structured questionnaires.

### Data quality control

To assure the quality of data, properly designed data collection instruments were prepared. Initially, the tools were prepared in the English version and then was translated to the local language: Amharic language with the help of language expert, and translated back to English to ensure consistency. Training for data collectors and supervisors that include a briefing on the general objective of the study, and discussing the contents of the questionnaire was given. Before data collection, pre-testing was conducted in a 5% population among pregnant mothers with a natural planned conception attending ANC service at Birbir health center. The overall activity of data collection was supervised and coordinated by the principal investigator. The collected data were reviewed and checked for completeness before data entry.

### Data processing and analysis

Data was entered into Epi-data software version 4.4.2.1 and then exported to SPSS version 25 statistical package for analysis. Descriptive statistics were done and summarized by tables, frequencies, graphs, median, proportion, and interquartile range.

Chi-square was conducted to identify the association between sub-fecundity and each categorical independent variable, in sequence. As a result, variables that violated the chi-square assumption were not transferred to logistic regression. Variables failed chi-square assumptions test were: medical cases (HIV for both mother and partner and DM cases) before the current pregnancy and mothers’ cigarette smoking before the current pregnancy.

The binary logistic regression model was fitted to identify factors associated with sub-fecundity after checking assumptions. Variables removed from the models due to multi co-linearity by co-linearity matrix during multi-variable binary logistic regression were: partner drinking alcohol due to highly co-linearity with mother’s alcohol consumption (>0.7), mothers’ occupational status due to highly co-linearity with mother’s working hours(>0.9) and partner’s working hour due its highly co-linearity with partner’s occupation (> 0.9) before the current pregnancy.

Bivariate logistic regression analysis was performed between dependent and each of the independent variables, in sequence. Variables having a p-value of <0.25 in bi-variable logistic regression were a potential candidate for multivariable logistic regression analysis to control confounders in regression models. Variables having a p-value of less than 0.05 in the multivariable logistic regression model were considered as statistically significant. The final model was checked for fitness using Hosmer and Lemeshow (p-value 0.39). The strength of association between the outcome variable and independent variables were reported by using the adjusted odds ratio with 95% CI.

### Ethical considerations

This study was carried out after obtaining ethical clearance from Arba Minch University (AMU), College of Medicine and Health Sciences institutional research ethics review board. Formal letters were submitted and permission was sought from the hospital and health centers before conducting the study. Written consent was obtained from the mothers, the study participants. Similarly, no personal identifiers were used to collect the data, to maintain the confidentiality of the information and privacy.

## Results

### Socio-demographic characteristics

In this study, five hundred thirty-four mothers were involved in the study and making a response rate of 99%. The mean ± SD age of the respondents before the current pregnancy was 27 ± 4.968 years and 484(90.6%) were married. Participants attended college and above were 186(34.8%). Participants working hour’s ≤ 40 per week before the current pregnancy were 210(39.3%). The average monthly income of families before the current pregnancy > 6000 ETB were 178(33.3) (Table 1).

**Table 1:** Socio-demographic characteristics of mothers with natural planned conception attended ANC service at AMHFs, 2020.

Variables	Categories	Frequency (n = 534)	Percent (%)
Mother’s age (years)	19–24	216	40.4
	25–29	163	30.5
	30–35	155	29.0
Partner’s age (years)	24–29	216	40.4
	30–34	145	27.2
	35–40	173	32.4
Mother’s educational status	No formal education	113	21.2
	Primary education	123	23.0
	Secondary education	112	21.0
	College and above	186	34.8
Mother’s working hours average per week (hours)	Housewife*	189	35.4
	14–40	210	39.3
	41–60	135	25.3
Mother’s occupation	Housewife	189	35.4
	Merchant	126	23.6
	Government employee	127	23.8
	Daily laborer	27	5.1
	Others*	65	12.2
Partner’s educational status	No formal education	53	9.9
	Primary education	112	21.0
	Secondary	121	22.7
	College and above	248	46.4
Partner’s working hour average per week before the pregnancy (hours)	Farmer*	53	9.9
	11–40	207	38.8
	41–62	274	51.3
Partner’s occupation before the pregnancy	Farmer	53	9.9
	Merchant	130	24.3
	Government employee	208	39.0
	Daily laborer	65	12.2
	Others*	78	14.6
Average monthly income of Family before the pregnancy (ETB)	≤ 2000	98	18.4
	2001–4000	126	23.6
	4001–6000	132	24.7
	>6000	178	33.3
Duration of months live together before the pregnancy (months)	1–60	311	58.2
	61–120	157	29.4
	>120	66	12.4

Others*: privet worker, student, car driver, religion leader. Housewife*: Those working only domestic work, which is difficult to calculate working hours. Studies used it instead of calculating working hours. Farmer*: It is used instead of calculating working hours; it is difficult to calculate working hours.

### Substances use related factors

Among the participants, 394(73.8%) drink 1–3 cups of coffee per day before the current pregnancy. The majority of the participants’ partners 397(74.3%) drink 1–3 cups of coffee per day before the current pregnancy. Only 92(17.2%) of mothers drink alcohol. Of the total sample, mothers, that have the habit of chewing khat were 53(9.9%) and 91(17.0%) of the mothers’ partners chewing chat before the current pregnancy. Likewise, only 6(1.1%) of mothers smoke cigarettes, while 116(21.7%) the mothers’ partners smoke cigarettes per day before the current pregnancy (Table 2).

**Table 2:** Substances use related factors of mothers with natural planned conception attended ANC service at AMHFs and their partners, 2020.

Variables	Categories	Frequency (n = 534)	Percent (%)
Mother’s coffee drinking before the pregnancy (cups/day)	1–3	394	73.8
	≥4	64	12.0
	none	76	14.2
Partner’s coffee drinking before the pregnancy (cup/day)	1–3	397	74.3
	≥4	63	11.8
	None	74	13.9
Mother’s alcohol drinking before the pregnancy	Yes	92	17.2
	No	442	82.8
Partner’s alcohol drinking before the pregnancy	Yes	145	27.2
	No	389	72.8
Mother’s khat chewing before the pregnancy	Yes	53	9.9
	No	481	90.1
Partner’s khat chewing before the pregnancy	Yes	91	17.0
	No	443	83.0
Mother’s cigarette smoking before the pregnancy	No	528	98.9
	Yes	6	1.1
Partner’s cigarette smoking before the pregnancy	Yes	116	21.7
	No	418	78.3

### Reproductive health related factors

Among the participants, majority 361(67.6%) of them were multi gravid. Regarding abortion only 40 (7.5%) of them faced abortion before the current pregnancy. participants who practice a Coital frequency of less than 2 days per week were 44 (8.2%). The respondents who experienced irregular menses before the current pregnancy were 119(22.3%). Among the participants, the majority of them were using injection contraceptive method 176(33.0%), followed by 95(17.8%) implants before the current pregnancy (Table 3).

**Table 3:** Reproductive health-related factors of mothers with natural planned conception attended ANC service at AMHFs, 2020.

Variables	Categories	Frequency (n = 534)	Percent (%)
Abortion	Yes	40	7.5
	No	494	92.5
Menses regularity before the pregnancy (days)	Regular(21–35)	415	77.7
	Irregular(<21 or >35)	119	22.3
Gravidity	1 (Prim gravid)	173	32.4
	>1 (Multi gravid)	361	67.6
Parity	<1	184	34.5
	≥1	350	65.5
Coital frequency before the pregnancy (coital/week)	1	44	8.2
	2–3	314	58.8
	>3	176	33.0
Type of contraceptive used before the pregnancy	None	154	28.8
	Condom	22	4.1
	OCP	65	12.2
	Injection	176	33.0
	Implant	95	17.8
	IUD	22	4.1

### Medical related characteristics

Four hundred eighty-eight (91.4%) participants knew their HIV status before the current pregnancy. Among these, only 2 (0.4%) of them were positive and both of them were sub-fecund and 486(91%) of them were negative. The rest 46(8.6%) of them not knew their status before the conception. Regarding medical problem, 15(2.8%) of them became diabetes mellitus (DM) patients before the pregnancy and 13(2.4) of the participants’ partners became DM patients before the current pregnancy. All the partners who had been DM patients 13(100%) were sub-fecund (Table 4).

**Table 4:** Medical-related problems of mothers with natural planned conception attended ANC service at AMHFs, 2020.

Variables	Categories	Frequency (n = 534)	Percent (%)
Mother’s HIV status before the pregnancy	Positive	2	.4
	Negative	486	91.0
	Unknown	46	8.6
Partner’s HIV status before the pregnancy	Positive	2	.4
	Negative	456	85.4
	Unknown	76	14.2
Mother’s medical case before the pregnancy	DM	15	2.8
	Unknown	7	1.3
	None	512	95.9
Partner’s medical case before the pregnancy	DM	13	2.4
	Unknown	55	10.3
	None	466	87.3

### Waiting time to pregnancy

**Fig 1:**
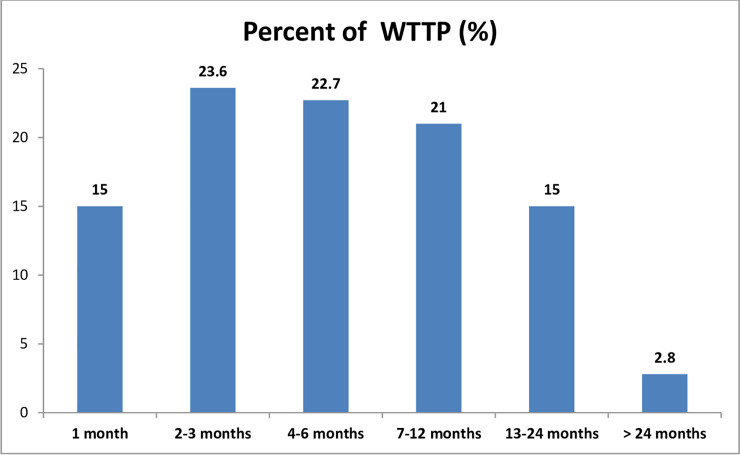
Waiting time to pregnancy of mothers with natural planned conception attended ANC service at AMHFs, 2020.

In this study, mothers with sub-fecundity status was 17.8% (95% CI:14.8%-21.3%). The median time of waiting months to get pregnancy was 5 months with an inter-quartile range of 8. Waiting time to pregnancy within the first months was 15%, WTTP within 6 months was 61.2%, within 12 months 82.2%, and waiting time to pregnancy greater than 12 months was 17.8% (Fig 1).

### Factors associated with sub-fecundity

Bi-variable logistic regression was conducted to identify candidate variables with a p-value < 0.25. The following variables were identify as a candidate for Multivariable logistic regression. Mother’s age, partner’s age, mother’s educational level, partner’s education level, duration of months live together, partner’s occupation, monthly income of a family, mother’s coffee-drinking per day, mother’s drinking alcohol, partner’s smoking cigarette, abortion, mother’s working hour, partner’s coffee-drinking per day, type of contraceptive used, coital frequency and menses regularity were entered to multivariable binary logistic regression to identify the possible association with sub-fecundity.

However, mother’s age, partner’s age, mothers’ drinking coffee per day before the current pregnancy, coital frequency before the current pregnancy and menses regularity before the current pregnancy were significantly associated factors with sub-fecundity status on multivariable logistic regression analysis (Table 5).

**Table 5:** Factors associated with sub-fecundity for mothers with natural planned conception attending ANC service at AMHFs, 2020.

Variables		Sub-fecundity status		COR(95% CI)	AOR(95% CI)	p-value
		Yes	No			
		n(%)	n(%)			
Mother’s age (years)	19–24	27(12.5)	189(87.5)	1	1	
	25–29	24(14.7)	139(85.3)	1.21(0.67–2.18)	1.33(0.63–2.82)	.453
	30–35	44(28.4)	111(71.6)	2.78(1.63–4.73)	2.54(1.18–5.48)	.017*
Partner’s age (years)	24–29	26(12.0)	190(88.0)	1	1	
	30–34	19(13.1)	126(86.9)	1.10(0.59–2.08)	0.94(0.41–2.14)	.884
	35–40	50(28.9)	123(71.1)	2.97(1.76–5.02)	2.20(1.01–4.75)	.046*
Mother’s education	No formal education	16(14.2)	97(85.8)	1	1	
	Primary education	17(13.8)	106(86.2)	0.97(0.47–2.03)	0.87(0.35–2.20)	.771
	Secondary	23(20.5)	89(79.5)	1.57(0.79–3.16)	1.25(0.48–3.24)	.651
	College & above	39(21.0)	147(79.0)	1.61(0.85–3.04)	1.80(0.65–5.02)	.261
Working hour/week (hrs)	Housewife	31(16.4)	158(83.6)	1	1	
	14–40	33(15.7)	177(84.3)	0.95(0.56–1.62)	1.04(0.45–2.39)	.931
	41–60	31(23.0)	104(77.0)	1.52(0.87–2.65)	1.89(0.83–4.29)	.129
Partner’s education	No formal education	5(9.4)	48(90.6)	1	1	
	Primary education	16(14.3)	96(85.7)	1.60(0.55–4.63)	2.31(0.60–8.93)	.226
	Secondary	22(18.2)	99(81.8)	2.13(0.76–5.99)	1.96(0.49–7.75)	.340
	College & above	52(21)	196(79.0)	2.55(0.97–6.72)	2.14(0.49–9.35)	.314
Partner’s occupation	Farmer	12(22.6)	41(77.4)	1	1	
	Merchant	14(10.8)	116(89.2)	0.41(0.18–0.96)	0.29(0.09–1.01)	.051
	Govn’t employee	44(21.2)	164(78.8)	0.92(0.44–1.89)	0.78(0.22–2.84)	.711
	Daily laborer	10(15.4)	55(84.6)	0.62(0.25–1.58)	0.54(0.16–1.82)	.318
	Other	15(19.2)	63(80.8)	0.81(0.35–1.91)	0.45(0.13–1.62)	.223
Average monthly income (ETB)	>6000	37(20.8)	141(79.8)	1	1	
	4001–6000	24(18.2)	108(81.9)	0.85(0.48–1.50)	0.90(0.43–1.90)	.783
	2001–4000	19(15.1)	107(84.9)	0.68(0.37–1.24)	0.99(0.44–2.26)	.999
	≤ 2000	15(15.3)	83(84.7)	0.69(0.36–1.33**)**	1.08(0.38–3.07)	.893
Live together (months)	≤ 60	48(15.4)	263(84.6)	1	1	
	61–120	25(15.9)	132(84.1)	1.04(0.61–1.76)	0.72(0.35–1.49)	.381
	> 120	22(33.3)	44(66.7)	2.74(1.51–4.98)	1.54(0.65–3.61)	.324
Mother’s drinking coffee (cups/day)	None	11(14.5)	65(85.0)	1	1	
	≥4	47(37.9)	77(62.1)	3.61(1.73–7.52)	2.93(1.14–7.53)	.026*
	1–3	37(11.1)	297(88.9)	0.74(0.36–1.52)	0.54(0.22–1.35)	.186
Partner’s coffee drinking (cups/day)	None	8(10.8)	66(89.2)	1	1	
	1–3	66(16.6)	331(83.4)	1.65(0.75–3.59)	1.15(0.43–3.07)	.777
	>3	21(33.3)	42(66.7)	4.13(1.68–10.16)	2.87(0.90–9.21)	.076
Mother’s alcohol drinking	No	74(16.7)	368(83.3)	1	1	
	Yes	21(22.8)	71(77.2)	1.47(0.85–2.54)	0.97(0.49–2.05)	.992
Partners cigarette smoking	No	61(14.6)	357(85.4)	1	1	
	Yes	34(29.3)	82(70.7)	2.43(1.50–3.94)	1.72(0.91–3.27)	.098
Abortion	No	85(17.2)	409(82.8)	1	1	
	Yes	10(25.0)	30(75.0)	1.60(0.76–3.41)	1.42(0.56–3.64)	.460
Menses regularity (days)	Regular	54(13.0)	361(87.0)	1	1	
	Irregular	41(34.5)	78(65.5)	3.51(2.19–5.65)	3.79(2.01–7.14)	.0001*
Coital frequency (days/week)	>3	30(17.0)	146(83.0)	1	1	
	2–3	45(14.3)	269(85.7)	0.81(0.49–1.35)	0.99(0.53–1.86)	.983
	1	20(45.5)	24(54.5)	4.06(1.99–8.26)	3.65(1.47–9.05)	.005*
Contraceptive used	None	19(12.3)	135(87.7)	1	1	
	Condom	7(31.8)	15(68.2)	3.32(1.20–9.17)	3.36(0.93–12.19)	.066
	OCP	13(20.0)	52(80.0)	1.78(0.82–3.85)	1.71(0.62–4.71)	.300
	Injection	35(19.9)	141(80.1)	1.76(0.96–3.23)	1.81(0.79–4.12)	.159
	Implant	16(16.8)	79(83.2)	1.44(0.70–2.96)	1.65(0.65–4.19)	.289
	IUD	5(22.7)	17(77.3	2.09(0.69–6.32)	0.87(0.19–3.98)	.860

* Significant association at a p-value of <0.05, 1: set as a reference group, COR- crude odd ratio, AOR -adjusted odd ratio, CI- confidence interval

## Discussion

This study was conducted to assess the proportion of sub-fecundity and associated factors among mothers with natural planned conception attending ANC service. The result showed that the proportion of sub-fecundity with a 95% confidence interval was 17.8% (14.8%-21.3%). Factors contributed to sub-fecundity in this study were mothers’ age before the current pregnancy, partners’ age before the current pregnancy, coffee drinking ≥ 4 cups per day before the current pregnancy, coital frequency of 1 day per week before the current pregnancy and irregular menses before the current pregnancy.

The proportion of sub-fecundity status in this study was consistent with the finding of study conducted in Addis Ababa (18.3%), southern Spain (17.58%), and European regions (16.1%) [8, 16, 32]. The proportion of sub-fecundity reported in this study was higher than the studies Conducted in Palestine village (13.4%) and German (10.4%) [11, 33]. The possible reason for this difference might be variation in the study population. Eighty-three percent (83%) of the study participants in Palestine village were in the age range of ≤ 24 years, while only 40.1% of the participants were in the age range of ≤ 24 in this study. Beside this the difference might be due to lifestyle difference of the study population that might be the contribution for this different percent of the sub-fecundity outcomes.

The proportion of sub-fecundity reported in this study was lower than the studies conducted in South Africa (22%), a demographic health survey of 12 countries (25%), and a multi-center study of Asian countries (45%) [3, 34, 35]. This might be due to the variation of study participants; The DHS study and Asian study was a multi-center study which included population with different socio-demographic characteristics and also the DHS study was conducted by including 10.1% HIV patients, which might be the possible reason of prolonged to conceive. Whereas the study in South Africa was only among 3 specific workers, while the current study was conducted among all mothers with different occupational status and the same catchment area mothers attended ANC service in AMHFs only.

In this study, the age of the mothers before the pregnancy was significantly associated with sub-fecundity. It revealed that mothers aged ≥30 years were 2.54 times more likely to be sub-fecund than mothers aged <25 years. This finding is in line with prior studies conducted in Denmark, Swedish, low to high-income countries multicenter study and studies conducted in Addis Ababa [8, 18, 29, 36], suggesting that as age advanced, the probability to get pregnancy greater than 12 months are more likely higher than younger age groups. It is the matter of fact that the fecundity status of woman declines gradually over the woman’s life span [37]. Owing to the biologically unavoidable fate of aging, the functions of cells of the ovarian, oviduct, uterine and immune systems are also affected.

The participants’ partners’ age before the pregnancy was significantly associated with sub-fecundity. This study showed that partners aged ≥35 years were 2 fold times more likely to be sub-fecund than partners aged <30 years old. This study is consistent with the study conducted in the Netherlands, a survey conducted from low to middle-income countries and Dogon of Mali [18, 25, 38]. The possible reason for the consistency might be due to the decrement of quality and quantity of sperm production as the age advanced.

Coffee drinking ≥ 4 cups per day before the current pregnancy was significantly associated with sub-fecundity. Mothers who drank 4 or more cups of coffee per day before the current pregnancy were 3 fold times more likely to be sub-fecund than mothers did not drink coffee. This is supported by studies conducted in the UK, European multicenter, and Addis Ababa [8, 39, 40]. The reason might be caffeine containment of coffee which disturbs ovulation and menstrual characteristics during high coffee intake per day. Scholars suggested that Caffeine is an adenosine receptor antagonist that may influence fecundity by affecting ovulation and menstrual characteristics, while a low amount of coffee intake has no such an adverse outcome.

A contrary to study conducted in the Netherlands [41], suggested that drinking a high amount of coffee reduces waiting time to pregnancy. The possible explanation might be coffee contents of caffeine and sample size. The reason high coffee affects fecundity is that, its caffeine content. When the concentration of coffee taken is diluted the amount of caffeine also reduced. Thus, the result of this study might be associated with coffee concentration. The second reason might be sample size, as the study sample size was 259 while in other studies > 400; due to the small sample size, the result might be unrepresentative of the source population which can result in an inappropriate outcome.

The frequency of sexual intercourse per week was significantly associated with sub-fecundity. Mothers who make sexual intercourse a day per week were 3.65 times more likely to be sub-fecund than mothers who make sexual intercourse >3 days per week. This is in line with the studies conducted in Utah [42]. The possible explanation might be due to fertilization. Fertilization occurs, when matured (ovulated) egg interacted with sperm. The maximum probability survival of egg after ovulation is 24 hours where the survival age of sperm after ejaculation into the women's reproductive organ is 24–72 hours. When sexual intercourse per week is one and less the probability of fertilization might be affected.

Menses regularity was one of determinate factor for sub-fecundity. Mothers who had irregular menses before the current pregnancy were approximately 4 fold times more likely to be sub-fecund than mothers who had regular menses. This study is consistent with prior studies in Addis Ababa, USA and Sweden [8, 36, 43]. The possible explanation for this might be due to ovulation. Fertilization is effected by the time of ovulation. When sexual intercourse and ovulation mismatched the occurrence of conception might be delayed. In this study, as the explained sexual frequency was one of the factors associated with the sub-fecundity, when ovulation is prolonged or within a short time of ovulation, time of sexual intercourse 1day per week might be mismatched with ovulated egg to fertilize. The menstrual cycle is associated with ovulation of the matured egg. When the time of ovulation is mismatched the couples might be confused about the appropriate time of sexual intercourse to get the planned pregnancy.

## Conclusion

In general, this study found that a substantial proportion of mothers were sub-fecund. Factors those contributed to sub-fecundity were:- Mothers’ age ≥ 30 years before the current pregnancy, partners’ age ≥ 35 years before the current pregnancy, coffee drinking ≥ 4 cups per day before the current pregnancy, coital frequency 1 day per week before the current pregnancy and irregular menses before the current pregnancy.

Planning health education delivery method that informs couples, to conceive at early age, reducing coffee consumption per day, increasing days of coital frequency per week and provision of appropriate investigation method and treatment for mothers facing irregular menses to reduce sub fecundity should be the focused method to tackle sub-fecundity.

## Supporting information

S1 Dataset (CSV)
